# Do Patients After Chondrosarcoma Treatment Have Age-appropriate Bone Mineral Density in the Long Term?

**DOI:** 10.1007/s11999-016-4741-3

**Published:** 2016-02-16

**Authors:** Gerhard M. Hobusch, Thomas M. Tiefenboeck, Janina Patsch, Christoph Krall, Gerold Holzer

**Affiliations:** Department of Orthopaedic Surgery, Medical University of Vienna, Waehringer Guertel 18-20, 1090 Vienna, Austria; Department of Neuroradiology and Musculoskeletal Radiology, Medical University of Vienna, Vienna, Austria; Center for Medical Statistics, Informatics, and Intelligent Systems, Medical University of Vienna, Vienna, Austria

## Abstract

**Background:**

In long-term survivors of osteosarcoma and Ewing sarcoma treated with the addition of radio- and chemotherapy, low bone mineral density (BMD) and fractures have been observed, presumably resulting from these adjuvants. Because patients with chondrosarcoma usually are not treated with conventional adjuvant treatment, observation of low BMD in patients with chondrosarcoma presumably would be the result of other mechanisms. However, BMD in patients with a history of chondrosarcoma has not been well characterized.

**Questions/Purposes:**

The aim of our study was to address the following questions: (1) Do long-term survivors of chondrosarcoma have normal BMD and, if not, which factors contribute to low BMD? (2) Is there a greater risk of fracture and does the Fracture Risk Assessment Tool (FRAX^®^) score reflect fracture likelihood?

**Methods:**

All known patients with a history of chondrosarcoma treated at our institution before 2006 were identified. Of 127 patients believed to be alive at the time of this study, 30 agreed to participate in this study (11 females, 19 males; mean age at surgery, 39 ± 12 years; mean followup, 12 ± 5 years). With the data available, the 30 participants were not different from the 97 nonparticipants in terms of age, sex, BMI, tumor grade, tumor location (axial versus appendicular, lower extremity versus elsewhere), and use of any treatment known to influence osteopenia (chemotherapy, lower extremity surgery). BMD was measured and history of fractures was assessed using a questionnaire. The patients´ BMD measurements in this study were sex- and age-matched with a normative sex- and age-categorized reference population reported by Kudlacek et al. Associations were tested by univariate regressions and ANOVAs of all measures of BMD and eligible oncologic and demographic factors.

**Results:**

Eighteen of 30 (60%) patients had a pathologic BMD according to the WHO dual-energy x-ray absorptiometry definition, 15 (50%) had osteopenia, and three (10%) had osteoporosis. T-scores in the study cohort were lower than reference values for the femur neck (mean difference, 0.64; 95% CI, 0.27–1.01; p < 0.0015), but not for the spine (mean difference, 0.39; 95% CI, −0.06 to 0.84; p = 0.09). Thirteen patients (45%) reported a history of fractures not distinguishing between low and high impact. The incidence of fractures was 2.8 greater than expected from a comparison with a published microcensus survey of the Austrian population. No effect of the FRAX^®^ score on fracture risk could be identified (p = 0.057).

**Conclusions:**

Long-term survivors of chondrosarcoma appear to be at greater risk for having low BMD develop than the healthy population. Although these results are preliminary and based on a very small sampling of patients, if they can be confirmed in larger studies, BMD assessment by dual-energy x-ray absorptiometry might be considered as these patients are followed posttreatment by sarcoma care units. The reasons for low BMD still must be elucidated.

**Level of Evidence:**

Level IV, prognostic study.

## Introduction

Patients with solitary chondrosarcomas, the second most common primary malignant bone tumor, are treated primarily by surgical resection because of lack of response to conventional radio- and chemotherapy [37]. Chemotherapy almost never is used in patients with chondrosarcoma, and radiation rarely is used, other than in patients with tumor recurrence or with marginal resection borders. By contrast, primary malignant bone tumors, including osteosarcoma and Ewing sarcoma, are treated using wide resection and (neo)adjuvant chemotherapy. Despite the advantages of chemo- and radiotherapy to patients’ survival, there is growing knowledge of side effects, including osteotoxic ones [2, 13, 15, 23, 34]. Low bone mineral density (BMD) has been observed in survivors of leukemia [1, 11], osteosarcoma [15, 34], and Ewing sarcoma [13, 34] after receiving chemotherapy. Decreased BMD in patients with bone sarcomas seems to be multifactorial in its genesis, and potential factors other than chemotherapy such as surgical treatment of patients, partial weightbearing periods, and long rehabilitation may contribute to low BMD in patients with bone sarcomas.

Less is known about chondrosarcoma and BMD. Patients with chondrosarcoma usually are not treated with adjuvant treatments, therefore if patients with chondrosarcoma are at risk of having low BMD develop, which to our knowledge has not been studied, this presumably would be the result of other mechanisms. Furthermore, pathologic fractures can occur at the time of presentation [6, 43] in chondrosarcomas in approximately 17% to 18% of patients [7, 36], but it is not known whether fracture risk is greater in patients with chondrosarcoma than in the healthy population, or whether that risk might be attributable to low BMD in patients with chondrosarcoma.

The aim of our study was to address the following questions: (1) Do long-term survivors of chondrosarcoma maintain normal BMD and, if not, which factors contribute to low BMD? (2) Is there a greater risk of fractures and does the Fracture Risk Assessment Tool (FRAX^®^) [18] score reflect fracture likelihood?

## Patients and Methods

Between 1971 and 2006, 249 patients with chondrosarcomas at different locations received a diagnosis and were treated at our department. One hundred eleven of these patients already had died of disease at the time of our study. Eleven patients had received chemotherapy for different reasons and therefore were excluded from this study. The study was approved by the institutional review board (EK 373/2009) and conducted according to the Helsinki Declaration. Informed consent was obtained from all participants before inclusion in the study.

Of the 127 patients who were informed by mail, 30 agreed to participate and were included in the study. This group consisted of 19 men and 11 women with a mean age of 39 years (SD, 12; range, 17–69 years) at the time of surgery and mean followup of 12 years (SD, 5; range, 5–24 years). A questionnaire was used to obtain demographic and clinical data. Oncologic data were taken from the prospective local tumor registry and supplemented by thorough chart reviews. Tumors were histologically graded as G1 to G3 by experienced bone pathologists (MS-K, IA, SL) at our institution. Grade G1 tumors were seen in 19 (63%) patients, G2 in eight (27%), and G3 in three (10%). The differentiation of a G1 chondrosarcoma was done when at least all four criteria (cellularity, matrix changes, binuclearity, and nuclear atypia) were fulfilled. The pathology reports from the treatment of these 30 patients were used in this study.

With the data available, the 30 participants were not different from the 97 nonparticipants, who were lost to followup, in terms of age, sex, BMI, tumor grade, tumor location (axial versus appendicular), and use of any treatment known to influence bone health (lower extremity surgery). In the nonparticipants, there were more lower extremity tumors versus tumors elsewhere (Table 1).

**Table 1:** Demographics of survivors with chondrosarcoma according to their DEXA-derived bone mineral density

Variable	Healthy	Osteopenia	Osteoporosis	Study participants (total)	Patients lost to followup	p value
	(n = 12)	(n = 15)	(n = 3)	(n = 30)	(n = 97)	
Demographic						
Sex (F/M)	5/7	5/10	1/2	11/19	41/56	0.67
Age (at time of surgery)^†^	34 ± 9	41 ± 13	47 ± 21	39 ± 13	40 (10–86)	0.67
Age (at time of DEXA)^†^	46 ± 11	52 ± 13	57 ± 19	51 ± 13		
Followup^†^	14 ± 6	12 ± 5	11 ± 3	13 ± 5		
BMI (kg/m^2^)^‡^	27 ± 4	26 ± 5	23 ± 2	26.2 ± 5	25.7 (17–35)	0.74
Physical functioning (SF-36)^‡^	66 ± 33	58 ± 34	77 ± 20	63 ± 32		0.6
Tumor grade (available for 88 patients; missing for 9 patients)						
G1	9	7	3	19	46	0.39
G2	2	6	0	8	29	1.0
G3	1	2	0	3	13	0.75
Metastasis	0	1	0	1		
Local recurrence	2	1	0	3		
Localization of tumor (97 patients)						
Tibia/fibula	2	2	0	4	10	0.73
Femur	1	2	1	4	23	0.3
Pelvis	1	3	0	4	21	0.43
Scapula	2	0	0	2	3	1.0
Humerus	3	2	0	5	10	0.34
Finger	1	2	2	5	23	0.64
Ribcage	1	3	0	4	6	0.12
Spine	1	1	0	2	1	0.13
Axial				6	31	0.25
Appendicular (extremities, scapula, and pelvis)				24	66	0.25
Lower extremity				12	61	0.03
Elsewhere				18	35	0.03
Surgical method						
Curettage	5	4	2	11	27	0.37
Resection	4	4	0	8	34	0.5
Endoprosthetic reconstruction	3	4	0	7	29	0.64
Amputation	0	3	1	4	7	0.28
Lower extremity surgery				12	61	0.03

Ranges are presented in parentheses; ^†^ mean in years; ^‡^mean; DEXA = dual-energy x-ray absorptiometry; all patients were Caucasian and were free of diabetes, obesity, or other metabolic disorders other than oncologic disease.

All patients had surgery at our institution. The common surgical approach of limb salvage was an excision or resection of the tumor with or without reconstruction by using a mega-endoprosthesis. Nineteen patients (63%) either had excisions or resections only, seven (23%) received an endoprosthetic reconstruction after resection of the tumor (Kotz Modular Femoral Tibial Replacement [KMFTR^®^]; Stryker Orthopaedics, Mahwah, NJ, USA), and four (14%) had an amputation. Resection margins were intralesional in 10 patients, marginal in one, and wide in 19. Tumors were located in the proximal humerus (n = 5), proximal femur (n = 2), distal femur (n = 2), pelvis (n = 4), tibia or fibula (n = 4), scapula (n = 1), rib cage (n = 5), spine (n = 2), and long finger bones (n = 5).

### Densitometric Technique

BMD of the lumbar spine (L1–L4) and of the proximal femur (total femur, femoral neck) of the nonoperated side was assessed by dual-energy x-ray absorptiometry (DEXA) (Hologic Discovery^TM^ A, Hologic Explorer^TM^; Hologic Inc, Bedford, MA, USA) (S/N 45313; Lunar^®^ Prodigy; GE Medical Systems, Munich, Germany). BMD results are expressed in terms of SD from a reference population (T-scores) and from a healthy, age- and sex-matched reference population (Z-score). To correct for the different facilities, BMD values were calculated for Lunar^®^ Medical Systems according to Genant et al. [9]. Patients were classified according to the WHO guidelines [35] in three groups: BMD values greater than −1 were considered normal, between −1 and −2.5 were classified as osteopenic, and less than −2.5 were considered osteoporotic [22].

An online fracture risk assessment tool [18] was used to calculate the FRAX^®^ [21].

### Laboratory Examination

Routine standard laboratory parameters like blood cell counts, electrolytes including serum calcium and phosphate, and bone turnover markers like osteocalcin, bone-specific alkaline phosphatase, parathyroid hormone, N-terminal propeptide of type I collagen, and the crosslinked C-telopeptides of type I collagen, calcitonin, thyroid, and sexual hormones were assessed for all 30 patients.

### Physical Function

All 30 patients had a physical examination and were evaluated according to Enneking et al. [8]. Physical ability was measured by Medical Outcomes Study SF-36 physical functioning subscale [5, 19]. The validity and reliability of the SF-36 has been established in patients with a history of cancer [3, 33].

### Normative Data

We used a published age-, sex-, and area-of-living matched control group for bone status [24]. The patients´ BMD measurements in our study were sex- and age-matched and compared by paired t-tests [24]. In addition, the incidence of osteopenia in our study population was compared with incidences reported in other studies (Table 2) [10, 29]. In our most prominent age group of patients (40–49 years), the incidence of osteopenia in men and women was 50%, whereas according to selected normative data 29% of 31% had osteopenia [27].

**Table 2:** Comparison of the prevalence of osteopenia

Study	Age groups (range in years)									
	20–24	25–29	30–34	35–39	40–44	45–49	50–54	55–59	60–64	65–69
Current study	50%		25%		50%		75%		71%	
Looker et al. (NHANES III) [29]	NA	NA	NA	NA	NA	NA	34%–49%			
Looker et al. (NHANES 2005–2006) [29]	NA	NA	NA	NA	NA	NA	32%–50%			
Guzmán Ibarra et al.* [10]	NA	NA	NA	NA	NA	18%	47%	44%	64%	53%
Larjiani et al.** [27]	13%–17%		8%–24%		29%–31%		42%––46%		47%–50%	

* Data presented for womens´ incidence of osteopenia; **data presented as lowest and highest value of womens´ and mens´osteopenia in femur neck and spine; NA = not available.

### Statistics

The patients´ BMD measurements in our study were sex- and age-matched with normative sex- and age-categorized data [24] and compared by paired t-tests. Univariate regressions of all measures of BMD on BMI, age at diagnosis, age at followup, and period between diagnosis and followup were performed. Correlation between fracture risk and T-scores was analyzed by logistic regression models. One-way ANOVA t-tests of all measures of BMD on sex, surgery, tumor grading, resection margins, and fractures were performed. The T-score depending on the FRAX^®^ was strongly right skewed, therefore this variable was transformed by taking the square root to obtain a more symmetric distribution and improve model fit. Owing to the explorative nature of the study, no correction for multiple hypothesis testing was done. All statistical calculations were performed with R 2.15.2 under R-studio (The R Project for Statistical Computing, Vienna, Austria).

## Results

### Bone Mineral Density

Overall 18 (60%) of the patients showed low BMD values. Three patients (10%; one female, two males) had osteoporosis, 15 (50%; five females, 10 males) had osteopenia, and 12 (40%; five females, seven males) had normal BMD (Table 3). T-scores in the study cohort were less than those of age- and sex-matched reference values for the femoral neck (mean difference, 0.64; 95% CI, 0.27–1.01; p = 0.0015), but not for the spine (mean difference, 0.39; 95% CI, −0.06 to 0.84; p = 0.09). Patients with higher BMI had higher T-scores of the lumbar spine (0.101; 95% CI, 0.01–0.19; p = 0.031), but not of the femoral neck (0.02; 95% CI, −0.07 to 0.12; p = 0.61) Age at diagnosis (−0.04; 95% CI, –0.07 to –0.01; p = 0.007) and age at followup (−0.37; 95% CI, −0.07 to − 0.01; p = 0.013) showed negative correlations with the T-score of the femoral neck. However, no correlation of T-scores of the lumbar spine was identified for age at diagnosis and for age at followup. The followup period, type of surgery, and physical ability/activity (SF-36 physical functioning) were not associated with T-scores of the femoral neck nor the lumbar spine. Physical ability in patients who had surgery of the upper limb (77 ± 27) compared with the lower limb (60 ± 27) showed no substantial difference (mean difference, 17; 95% CI, −10 to 45; p = 0.21). In addition, T-scores of the femoral neck in patients who had upper limb surgery (−0.99 ± 1.1) and lower limb surgery (−1.5 ± 0.78) showed no difference (mean difference, −0.5; 95% CI, −1.4 to 0.5; p = 0.27).

**Table 3:** Bone mineral density in survivors of chondrosarcoma

DEXA localization	Score	Normal bone			Osteopenia			Osteoporosis			p value
		Females (n = 5)	Males (n = 7)	Total number of patients (12)	Females (n = 5)	Males (n = 10)	Total number of patients (15)	Females (n = 1)	Males (n = 2)	Total number of patients (3)	
Total femur	T-score	0.6 ± 1.0	0.4 ± 0.7	0.5 ± 0.8	−0.6 ± 0.6	−1.1 ± 0.5	−0.9 ± 0.6	−2.3 ± 0.0	−2.0 ± 0.1	−2.1 ± 0.2	<0.001
	Z-score	1.0 ± 1.0	0.5 ± 0.8	0.7 ± 0.9	−0.1 ± 1.1	−0.6 ± 0.5	−0.4 ± 0.7	−1.5 ± 0.0	−1.5 ± 0.4	−1.5 ± 0.3	<0.001
	g/cm²	1.0 ± 0.1	0.9 ± 0.4	1.0 ± 0.3	0.9 ± 0.1	0.9 ± 0.1	0.9 ± 0.1	0.7 ± 0.0	0.7 ± 0.0	0.7 ± 0.0	0.18
Femoral neck	T-score	−0.3 ± 0.7	0.1 ± 0.9	0.0 ± 0.8	−1.6 ± 0.4	−1.7 ± 0.3	−1.7 ± 0.4	−2.9 ± 0.0	−2.3 ± 0.1	−2.5 ± 0.4	<0.001
	Z-score	0.4 ± 0.7	0.6 ± 0.8	0.5 ± 0.7	−0.9 ± 0.4	−0.8 ± 0.4	−0.8 ± 0.4	−1.8 ± 0.0	−1.4 ± 0.6	−1.5 ± 0.5	<0.001
	g/cm²	0.8 ± 0.1	0.8 ± 0.4	0.8 ± 0.3	0.7 ± 0.1	0.7 ± 0.1	0.7 ± 0.1	0.5 ± 0.0	0.6 ± 0.0	0.6 ± 0.1	0.13
L1–L4	T-score	−0.4 ± 1.1	0.1 ± 0.9	−0.1 ± 1.0	−1.0 ± 0.8	−1.0 ± 0.8	−1.0 ± 0.8	−3.2 ± 0.0	−2.8 ± 0.2	−2.9 ± 0.3	<0.001
	Z-score	0.3 ± 1.2	0.4 ± 1.0	0.3 ± 1.0	−0.2 ± 1.3	−0.4 ± 0.7	−0.3 ± 0.9	−2.0 ± 0.0	−2.2 ± 0.4	−2.1 ± 0.3	0.001
	g/cm²	1.0 ± 0.1	1.1 ± 0.1	1.1 ± 0.1	0.9 ± 0.1	1.0 ± 0.1	1.0 ± 0.1	0.7 ± 0.0	0.8 ± 0.0	0.8 ± 0.1	0.001

DEXA – dual-energy x-ray absorptiometry

### Fracture Risk

Thirteen patients reported a history of fractures (45%). Throughout the study population, the incidence of fractures per year was 0.034. In comparison, incidence rates in the Austrian reference population range between 0.010 and 0.018 fractures per year in the corresponding sex-specific age groups [41]. In an age and sex-matched sample from the population, a total of 4.7 fractures would be expected when accounting for the followup period for each patient. Fractures in the study population were not localized at typical osteoporotic fragility fracture sites (Table 4). BMD expressed by T-scores of the femoral neck (odds ratio [OR], 0.59; 95% CI, 0.25–1.21; p = 0.181) and lumbar spine (OR, 0.86; 95% CI, 0.44–1.62; p = 0.64) did not show effects on the incidence of fractures. The FRAX^®^ score did not show effects on the incidence of fractures (OR, 2.5; 95% CI, 0.97–6.54; p = 0.057). No substantial difference in SF-36 physical function score was seen between patients with fractures (60 ± 31) and those with no fractures (66 ± 33) (mean difference, 6; 95% CI, −19.11 to 31.78; p = 0.61).

**Table 4:** Fractures in survivors of chondrosarcoma

Variable	Number of fractures (males/females)			
	Normal BMD	Osteopenia	Osteoporosis	Total
	4 (4/0)	6 (3/3)	3 (2/1)	13 (9/4)
FRAX^®^ score (calculation included BMD)	0.7 (0–2)	3.9 (1–11)	6.7 (4–8)	3.5 (0–11)
Location of fracture				
Hand (carpus, phalanges)	2 (2/0)		2 (1/1)	4 (4/1)
Distal radius		2 (0/2)		2 (0/2)
Humerus		1 (0/1)		1 (0/1)
Tibia	1 (1/0)	2 (2/0)	1 (1/0)	4 (4/0)
Femur	1 (1/0)	1 (1/0)		1 (1/0)

FRAX^®^ = Fracture Risk Assessment Tool; BMD = bone mineral density.

## Discussion

Chondrosarcoma is the second most-frequent malignant bone tumor. The standard treatment of patients with chondrosarcoma is a wide resection or aggressive curettage for selected low-grade extremity chondrosarcomas only. Although other primary malignant bone tumors like osteosarcoma and Ewing sarcoma are treated with a multidisciplinary approach including chemotherapy and surgical resection, patients with chondrosarcoma do not show any clinical response to conventional adjuvant treatments. Low BMD and increased fracture rates after multimodal treatment of chemosensitive primary bone tumors are presumed to be osteotoxic side effects of chemotherapeutic treatments [2, 13, 15, 23]; however, other risk factors for low BMD in patients with a history of bone tumors may be present. To test the BMD of patients with chondrosarcoma, and because treatment of osteoporosis and osteopenia may be indicated once it is discovered, we wanted to assess whether patients with chondrosarcoma experience low BMD even in the absence of potentially osteotoxic chemotherapeutic treatment.

This study is limited because of the patients assumed to be alive, only 25% responded and agreed to participate. Survivors of malignant diseases may decline participation in studies for various reasons including general rejection of study participation or owing to professional commitments, not having time for followups, or relocation of residence. This poses a selection bias. However, because our tumor register’s demographic and oncologic data for the 97 patients lost to followup, including age, sex, BMI, tumor grade, tumor location (axial versus appendicular), and use of any treatment that could influence osteopenia (eg, chemotherapy), did not differ from the data of our study patients (Table 1), we believe our study patients reasonably represent the population of patients with this condition. However, there were more lower extremity tumors in the 97 nonparticipants, therefore you could argue that, if less mobility plays a role after treatment of lower extremity tumors, which potentially could lead to less BMD, then the nonparticipants would be even more prone to low BMD, and results would be even more powerful.

The tumors varied in size and by site with some lower extremity and some upper extremity lesions. In addition patients had many different types of surgical procedures, which potentially could affect BMD findings. With the small numbers available, we could not show that these issues were statistically related to BMD, but a study of larger groups of patients might show differences. Our patient population includes patients of differing ages, and with low- and high-grade tumors, major resections/reconstructions, and curettage. Our numbers are not large enough to look at differences between a high-grade chondrosarcoma of the lower extremity treated with megaprostheses and a small chondrosarcoma of the finger. Additional study is needed with a larger, more homogeneous population of patients before our findings can be generalized. Finally, our numbers are too small to perform a multivariate analysis to assess which are the most important factors predicting low BMD in the patients with chondrosarcoma; as such, confounding variables may have influenced some of our findings. No-difference findings could be a function of insufficient sample size.

Eighteen (60%) of 30 patients in our study had a pathologic BMD. T-scores in the study cohort were lower than reference values for the femoral neck (p < 0.001), but not for the spine (p = 0.11). Osteoporosis was seen in four patients. Surgical treatment of patients with bone sarcoma is followed by a long rehabilitation and periods with partial weightbearing. Patients often are restricted in their activities of daily living [32], physical activity [14], and sports activity [12, 25], which may influence BMD because of inactivity [17, 26, 28, 30]. Furthermore, in patients with chondrosarcoma, pathologic fractures occur at presentation of the disease [6, 43] in approximately 17% to 18% [6] and it is not known whether these patients continue to experience bone events once a chondrosarcoma has been diagnosed. Unlike patients with bone tumors which are treated with chemotherapy and radiation and are known to affect bone density [13, 15, 38], patients with chondrosarcoma generally do not receive these adjuvant treatments. Therefore, the reason for the lower-than-expected BMDs in patients with chondrosarcoma is not known However, abnormally low BMD in our patients was surprisingly common (60%; 18 of 30), and we did not detect any particular predictors of this apart from BMI, which exerted a protective effect. Morin et al. [31] discussed weight and body mass predicting low BMD in women 40 to 59 years old. In line with these data, the most powerful factor indicating low Z-scores of the femoral neck in our study was BMI. However, this appears to be an associated factor in patients with postmenopausal osteoporosis and in patients with a history of chemosensitive malignant bone tumors [13, 38, 40]. Although these long-term data did not reveal a correlation between sex hormones and bone status at the time of followup, there is some evidence that estrogen metabolism may be associated with the occurrence of chondrosarcoma and translational research has validated estrogen signaling a potential antitumor-therapeutic target [7]. Because sex hormones are involved in bone homeostasis as well, there could be a possible link. Another possibility is that the BMD findings may be “tumor-induced,” that is, related to a factor produced by the tumor, which is seen in certain types of tumors such as myeloma [42]. In these patients bone loss is mediated by various biologic factors produced by osteoblasts or by malignant plasma cells [42]. Tumor-induced bone loss also is seen with metastatic disease [4]. Future studies will need to determine whether there is any such tumor-related factor produced by chondrosarcoma cells.

A total of 45% of our patients (13 of 30) experienced fractures, and these fractures did not occur at typical osteoporotic fragility fracture sites. Two low-impact radius fractures were reported; the others may be considered high-impact fractures The incidence of fractures in our study population was higher than expected by a factor of 2.8 compared with a microcensus survey of the Austrian population [41]. Low BMD has been shown to be a good predictor for fractures in the elderly [20, 39]. Our study did not reveal an effect of low BMD on fractures in patients with a history of chondrosarcoma. There might be other reasons for fractures than low BMD, such as changes of the cortical bone, that cannot be assessed by DEXA [16]. In addition, no correlation between FRAX^®^ score and fracture risk was identified.

Our study showed an abnormal BMD in the majority of patients with chondrosarcoma. Patients with a history of chondrosarcoma appear to have low BMD of the proximal femur develop for reasons yet unknown and might have higher fracture rates than healthy age-related persons. The no-difference findings concerning low BMD in this series regarding age, different surgical techniques, tumor sites, and followup may be the result of insufficient sample size; alternatively, they may represent a yet-to-be defined tumor-related effect associated with chondrosarcoma, as seen in certain other tumors [4, 42]. As a consequence, physicians should be aware of the potential for low BMD in patients with chondrosarcoma and should consider evaluation and possible treatment of those with low BMD. Additional studies with larger numbers of patients with chondrosarcoma are necessary to confirm our findings, but our study may serve as a pilot study to further investigate the hypotheses of possible tumor-related bone loss in patients with sarcomas.
